# Diagnostic Contribution of the DSM-5 Criteria for Internet Gaming Disorder

**DOI:** 10.3389/fpsyt.2021.777397

**Published:** 2022-01-05

**Authors:** Tao Luo, Dan Wei, Jiangfan Guo, Maorong Hu, Xuelin Chao, Yan Sun, Qian Sun, Shuiyuan Xiao, Yanhui Liao

**Affiliations:** ^1^Department of Social Medicine and Health Management, Xiangya School of Public Health, Central South University, Changsha, China; ^2^Department of Psychology, The First Affiliated Hospital of Nanchang University, Nanchang, China; ^3^Department of Psychiatry, Jiangxi Mental Hospital, Nanchang, China; ^4^Publicity Division of Jiangxi Mental Hospital, Nanchang, China; ^5^National Institute on Drug Dependence, Peking University, Peking, China; ^6^Department of Psychiatry, Sir Run Run Shaw Hospital, Zhejiang University School of Medicine, Hangzhou, China

**Keywords:** internet gaming disorder, IGD, DSM-5, criteria, diagnostic, prevalence

## Abstract

**Background:** Internet gaming disorder (IGD) can have long-term severe consequences in affected individuals, especially adolescents and young people. Empirical studies of IGD using the DSM-5 criteria are still lacking. This study aimed to evaluate the contribution of specific criteria to the diagnosis of IGD based on the DSM-5 in the context of Chinese culture.

**Methods:** The Chinese version of the Internet Gaming Disorder Scale–Short Form (IGDS9-SF) was applied to investigate the prevalence of IGD in a general sample of 28,689 middle school students aged 12–19 years from two cities in China.

**Results:** The prevalence of IGD was 4.6% among this adolescent sample. The group of IGD students reported longer weekly gaming times and worse academic performance than the group of non-IGD students. Although “preoccupation” and “playing to escape” were the most frequently reported criteria, the conditional inference trees showed that “give up other activities,” ‘negative consequences,” and “continue despite problems” contributed most to the diagnosis of IGD based on the DSM-5.

**Conclusions:** The prevalence of IGD among Chinese adolescents (ages 12–19) was 4.6%. This study provides evidence for retaining or deleting specific diagnostic criteria by the DSM framework in the future.

## Introduction

Internet gaming disorder (IGD), an increasingly serious public health concern, can have long-term severe consequences (such as self-esteem problems, emotional distress, impaired executive control and cognitive function, and disrupted regional structural connectivity) in affected individuals, especially adolescents and young people (1–3). Therefore, the American Psychiatric Association (APA) included IGD in the appendix of the DSM-5 as a potential diagnosis. Referring to the diagnostic criteria for substance use disorders, the DSM-5 drafted diagnostic criteria for IGD and indicated that further research is warranted.

According to DSM-5, the clinical diagnosis of IGD as indicated by endorsing five (or more) of the following nine criteria: “(1) preoccupation with online/offline gaming (i.e., preoccupation); (2) experience of unpleasant symptoms when gaming is taken away (i.e., withdrawal); (3) the need to spend increasing amounts of time engaged in games (i.e., tolerance); (4) unsuccessful attempts to control participation in games (i.e., loss of control); (5) loss of interest in previous hobbies and entertainment as a result of, and with the exception of games (i.e., give up other activities); (6) continued excessive use of games despite knowledge of psychosocial problems (i.e., continuation); (7) deceiving family members, therapists, or others regarding the amount of gaming (i.e., deception); (8) use of games to escape or relieve negative moods (i.e., escape); and (9) jeopardising or losing a significant relationship, job, or education or career opportunity because of participation in games (i.e., negative consequences) (4).”

Many studies have also examined the validity of particular criteria in different cultural contexts. Rehbein et al. presented an early population-based evaluation study of the DSM-5 criteria. They found that the criteria “playing to escape” and “preoccupation” were less likely to predict IGD, whereas “give up other activities,” “tolerance,” and “withdrawal” were the most important predictor variables for IGD in Germany (5). Similarly, Király et al. conducted an online survey of Hungarian individuals and demonstrated that “preoccupation” and “playing to escape” provided limited information for the estimation of IGD severity (6). Both Besser et al. in Germany and Lemmens et al. in the Netherlands found that “playing to escape” has limited diagnostic power (7, 8). Ko et al. clinically assessed the presence of the nine criteria in China. They found that all DSM-5 criteria of IGD had good diagnostic performance except for “deception” and “escape”; the criteria “continuation” and “negative consequences” showed the best diagnostic accuracy (9).

More recently, the 11th version of the International Classification of Diseases (ICD-11) officially included gaming disorder as a mental disorder (10). Different from the DSM-5 framework, the ICD-11 framework eschewed the criteria of “tolerance” and “withdrawal” and applied a monothetic approach in which all criteria must be endorsed. The ICD-11 criteria involve (1) impaired control over gaming; (2) increasing priority given to gaming over other activities; (3) continuation of gaming despite the occurrence of negative consequences; and (4) resulting clinically significant distress or impairments in important areas of functioning (10).

The prevalence rates of IGD among adolescents range from 1.2 to 10% in Western countries and from 7.5 to 15% in Asian countries (8, 11, 12). China has the most gamers in the world, with nearly 1 billion gamers reported in 2021 (13), resulting in a high prevalence of IGD ranging from 2.1 to 17% (14–16). The estimated prevalence of IGD among Chinese adolescents diverges considerably across studies, which is partly due to differences in the assessment instruments used. Although overlap exists between the dimensions assessed with these instruments and the DSM-5 criteria for IGD, few studies have evaluated all nine DSM-5 criteria in the Chinese general population and estimated the IGD prevalence in a general adolescent sample.

The present study aimed to assess the discriminative validity of specific criteria based on the DSM-5 criteria of IGD and to determine the 12-month prevalence of IGD among Chinese adolescents.

## Methods

### Study Population and Procedure

Between November 2019 and January 2020, a cross-sectional study was conducted among middle schools in Weifang city of Shandong Province and Yingtan city of Jiangxin Province in China. We first randomly selected 17 high schools from these two cities and randomly selected 500 classes comprising 30,560 students from these high schools. Among all students selected, 306 were not in school at the time of the survey, so they were invited to complete the questionnaire at home. Finally, excluding 1,588 students who refused to participate in the research, a total of 28,972 students participated in the research, and the overall response rate was 94.80%.

The questionnaire mainly covered issues related to Internet use, including IGD, psychopathological status, family environment and school atmosphere. All students and their parents were informed that the purpose of the study was to understand adolescents' life situation, mental health status, and Internet use. The students and their parents were also told that if they were unwilling to participate in the survey, they could not participate; or if they were willing to discontinue participation in research, they could stop filling in the questionnaire at any time, which would not have any impact on their study and life. Then, informed consent was sent to each student's parents. After obtaining informed consent from parents, an electronic informed consent form was then obtained from each student. The survey was carried out in accordance with the Declaration of Helsinki. Ethical approval was obtained from the ethics committee of Jiangxi Mental Hospital of Nanchang University (No. 20190113).

### Measures

Socio-demographics such as sex and age were collected. Weekly game play was calculated as follows: (daily game play time on a week day × 5) + (daily game play time on a weekend day × 2).

#### Internet Gaming Disorder

The Chinese version of the Internet Gaming Disorder Scale–Short Form (IGDS9-SF) was administered to assess the DSM-5 criteria for IGD (17, 18). This instrument is a 9-item scale with each item representing a DSM-5 criterion. Each item was rated on a five-point Likert scale ranging from “1 = never” to “5 = very often.” A criterion was considered endorsed if the corresponding item was answered with “often” or “very often” (17). The suggested cut-off score for IGDS-9SF is 32 (19). In this study, the Cronbach's α was 0.92.

#### Academic Record

Academic records were assessed by asking participants the question “Based on the total scores of all subjects in your most recent final exam, how about your academic performance? 1 = very bad, 2 = bad, 3 = medium, 4 = good, or 5 = very good.”

### Statistical Methods

#### Missing Data

Among the participants, 28,689 (99.02%) students responded to all items of the questionnaire. The data of 283 other (0.98%) students were excluded from the analysis because of missing important information (without information for IGDS9-SF values).

#### Statistical Procedures

We calculated Cohen's kappa coefficients to assess the endorsement of specific criteria corresponding to IGD. Then, we used non-parametric conditional inference trees (C-Trees) to explore the contributions of specific criteria to the diagnosis of IGD. The predictive variables were age, gender, weekly gaming time, and nine IGD criteria, while the response variable was IGD diagnosis.

## Results

### Sample Characteristics and Game-Play-Related Behaviours

The demographic characteristics are shown in Table 1. The students ranged from 12 to 19 years old (15.61 ± 1.90), and 44.2% were male. Male students showed more gaming time per week than female students (8.27 ± 9.53 h vs. 4.70 ± 6.25 h) and higher IGDS9-SF scores (18.54 ± 7.99 vs. 13.54 ± 6.02).

**Table 1:** Sample characteristics and game-play-related behaviours (*n* = 28,689).

	**Total sample**	**Male students**	**Female students**	**Male students vs. female students**		
	**(*N* = 28,689)**	**(*n* = 12,674)**	**(*n* = 16,015)**	***t/Z/*χ*^2^***	***P***	**Effect size**
	**M (SD)**	**M (SD)**	**M (SD)**			
Age	15.61 (1.90)	15.55 (1.82)	15.65 (1.97)	*t* = −4.37	<0.001	Cohen's *d* = 0.05
Weekly gaming (hours)	6.31 (8.04)	8.27 (9.55)	4.70 (6.25)	*Z* = −48.10	<0.001	*r* = 0.28
IGDS9-SF score	15.74 (7.39)	18.54 (7.99)	13.53 (6.02)	*Z* = −60.74	<0.001	*r* = 0.36
Academic Record	3.05 (1.05)	3.14 (1.17)	2.98 (0.95)	*Z* = −14.05	<0.001	*r* = 0.08
	***N*** **(%)**	***N*** **(%)**	***N*** **(%)**			
**Middle school stage**						
Junior middle school	13,474 (46.97)	5,671 (44.75)	7,803 (48.72)	χ^2^ = 44.95	<0.001	ϕ = 0.04
Senior middle school	15,215 (53.03)	7,003 (55.25)	8,212 (51.28)			
Internet gaming disorder	1,327 (4.63)	995 (7.85)	332 (2.07)	χ^2^ = 535.36	<0.001	ϕ = 0.36

*IGDS9-SF, The nine-item Internet Gaming Disorder Scale–Short-Form*.

### Prevalence of Internet Gaming Disorder

The estimated 12-month prevalence of IGD was 4.63%. The prevalence of IGD was 7.85% among male students, which was higher than that among female students (2.07%) (Table 1). As shown in Table 2, the IGD students reported more weekly game time and lower academic records than the non-IGD students.

**Table 2:** Validation of IGD classification (*n* = 28,689).

	**IGD-students**	**Non-IGD students**	***Z***	***P***	***r***
Weekly game time	29.47 (10.40)	5.14 (5.98)	−60.03	<0.001	0.35
IGDS9-SF score	35.69 (3.44)	14.77 (6.04)	−62.06	<0.001	0.37
Academic Record	2.97 (1.33)	3.05 (1.04)	−2.42	<0.01	0.01

*IGDS9-SF, The nine-item Internet Gaming Disorder Scale–Short-Form; IGD, Internet Gaming Disorder*.

### Endorsement and Predictive Power of the DSM-5 Criteria

As shown in Table 3, “preoccupation” was the most endorsed criterion (15.63%), followed by “escape” (14.1%). The proportion of participants endorsing “tolerance,” “loss of control,” “give up other activities,” “deception,” and “negative consequences” ranged between 5 and 8%. The most rarely endorsed criteria were “continuation” (4.32%) and “withdrawal” (4.87%).

**Table 3:** Endorsement of DSM-5 IGD criteria (*n* = 28,689).

**Criterion**	**Mean**	**SD**	**Criterion (%)**	**Cohen's kappa**
Preoccupation	2.28	1.27	15.63	0.39
Withdrawal	1.60	0.93	4.87	0.41
Tolerance	1.76	1.04	7.55	0.42
Loss control	1.70	0.98	5.48	0.45
Give up	1.61	0.98	5.41	0.51
Continuation	1.61	0.94	4.32	0.45
Deception	1.52	1.00	6.63	0.37
Escape	2.06	1.23	14.10	0.36
Negative consequences	1.62	0.96	5.49	0.47

*IGD, Internet Gaming Disorder*.

The criterion “give up other activities” corresponded best to the DSM-5 IGD diagnosis (Cohen's κ = 0.51), and other criteria including “loss of control,” “continue despite problems,” and “negative consequences” corresponded well to the IGD diagnosis (Cohen's κ > 0.45).

**Figure 1:**
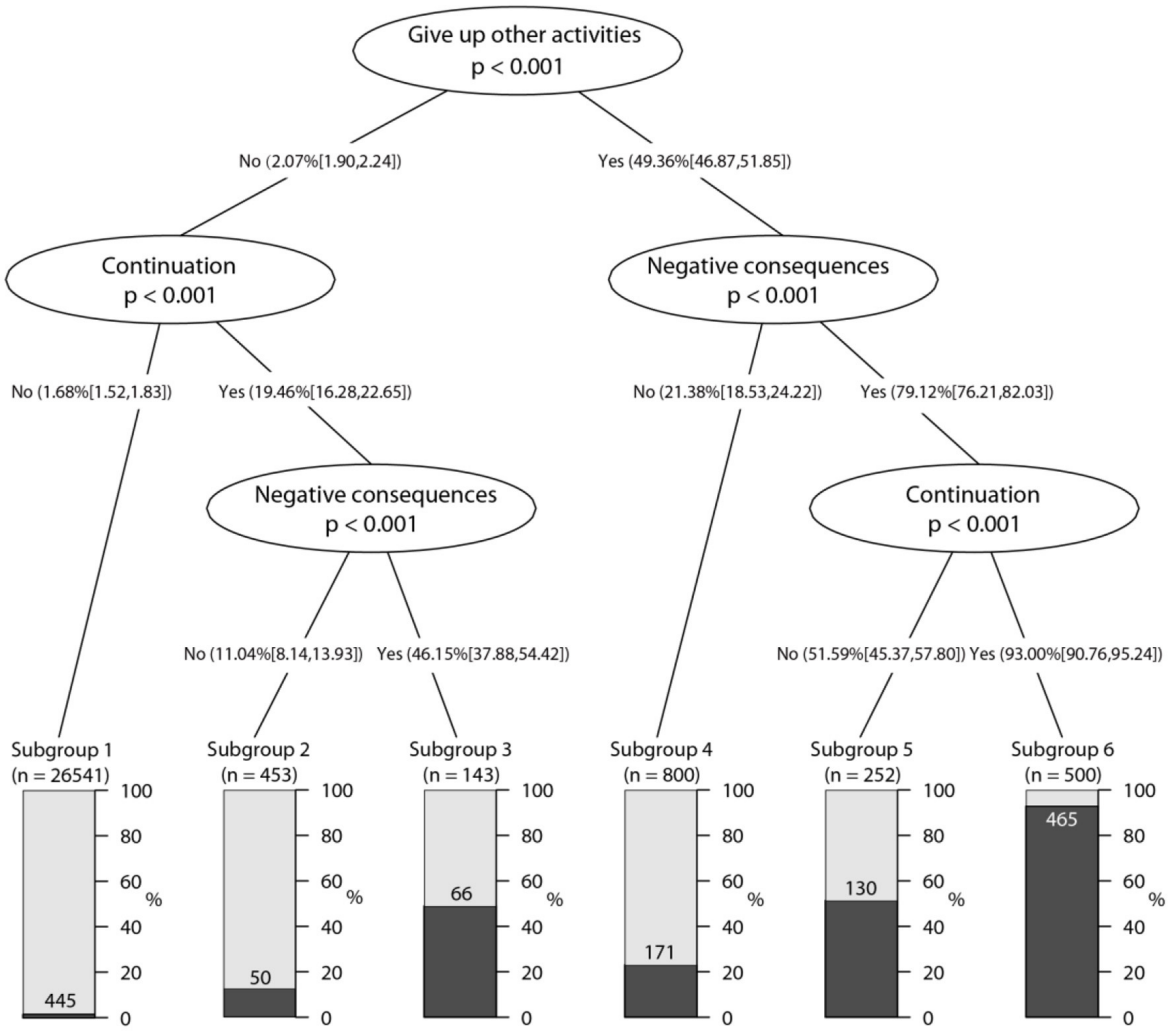
Conditional inference tree plot predicting DSM-5 IGD by diagnostic criteria, age, gender and gaming time (*n* = 28,689).

As illustrated in Figure 1, “give up other activities” was the single input variable with the highest predictive value for the DSM-5 IGD diagnosis, with a percentage of 49.36% (95% CL: 46.87, 51.85). For those adolescents who had endorsed “give up other activities,” if they also endorsed “continuation” and “negative consequences,” the percentage of adolescents (Subgroup 6) meeting the IGD diagnosis increased to 93.00% (95% CL: 90.76, 95.24); if they endorsed “negative consequences” but denied “continuation,” the IGD diagnostic rate was 51.59% (95% CL: 45.37, 57.80) (Subgroup 5); If they denied “negative consequences,” the IGD diagnostic rate was 21.38% (95% CL: 18.53, 24.22) (Subgroup 4).

As shown in the left part of Figure 1, a small percentage (2.07%, 95% CL: 1.90, 2.24) of adolescents meeting the IGD diagnostic standard did not endorse “give up other activities.” For those adolescents who had denied “give up other activities,” if they also denied “continuation,” the IGD diagnostic rate was 1.68% (95% CL: 1.52, 1.83) (Subgroup 1); if they endorsed “continuation” but denied “negative consequences,” the IGD diagnostic rate was 11.04% (95% CL: 8.14, 13.93) (Subgroup 2); and if they endorsed “continuation” and “negative consequences,” the IGD diagnostic rate was 46.15% (95% CL: 37.88%, 54.42%) (Subgroup 3).

### Comparison Variables of Impairment for IGD

There were 600 (2.10%) adolescents who met the criteria for IGD but did not endorse negative consequences, while 849 (2.96%) adolescents who did not meet the criteria for IGD but endorsed negative consequences. Given that functional impairment is necessary for a diagnosis of GD in the ICD-11 framework, we compared socio-demographics and game-play-related behaviours of adolescents with IGD endorse negative consequences, adolescents with IGD but did not endorse negative consequences, adolescents without IGD but endorse negative consequences, and adlescents without IGD did not endorse negative consequences.

As shown in Table 4, there were no significant differences in terms of gender and age among the group of IGD adolescents endorsed with negative consequences, IGD adolescents denied negative consequences, and non-IGD adolescents endorsed negative consequences; however, compared to the group of adolescents without IGD denied negative consequences, these three groups of adolescents tended to be male and older.

**Table 4:** Comparison variables of impairment for IGD (*n* = 28,689).

	**IGD students endorsed negative consequences** ***N* = 727**	**IGD students denied negative consequences** ***N* = 600**	**Non-IGD students endorsed negative consequences** ***N* = 849**	**Non-IGD students denied negative consequences** ***N* = 26 513**	***t*/χ^2^**	***P***	**Effect size**
	***N* (%)**	***N* (%)**	***N* (%)**	***N* (%)**			
Gender (male)	549 (75.52)_a_	446 (74.33)_a_	610 (71.85)_a_	11 069 (41.75)_b_	χ^2^ = 837.77^*^	<0.001	ϕ = 0.17
	***M*** **(SD)**	***M*** **(SD)**	***M*** **(SD)**	***M*** **(SD)**			
Age	15.98 (1.78)_a_	16.00 (1.61)_a_	16.05 (1.68)_a_	15.58 (1.92)_b_	*t* = 35.42	<0.001	Cohen's *d* = 0.03
Weekly game time	34.60 (7.17)_a_	23.25 (10.32)_b_	12.17 (10.32)_c_	4.92 (5.64)_d_	χ^2^ = 4,387.69^**^	<0.001	ϕ = 0.35
IGDS9-SF score	37.83 (3.23)_a_	33.11 (1.67)_b_	23.79 (4.91)_c_	14.49 (5.84)_d_	χ^2^ = 5,140.35^**^	<0.001	ϕ = 0.37
Academic Record	2.64 (1.30)_a_	3.36 (1.25)_b_	3.46 (1.15)_c_	3.04 (1.04)_d_	χ^2^ = 275.56^**^	<0.001	ϕ = 0.01

*IGDS9-SF, The nine-item Internet Gaming Disorder Scale–Short-Form; IGD, Internet Gaming Disorder*.

* *χ^2^ value was obtained by χ^2^ test*.

** *χ^2^ values were obtained by Kruskal-Wallis H test. Different subscript letters (a, b, c) in the same row reflect significant (p < 0.05) difference between the rates or means while same subscript letters in one row reflect non-significant difference between the rates or means according to χ^2^ test or Mann-Whitney U-test*.

In terms of game-play-related behaviours, adolescents with IGD endorsed negative consequences reported the most amount of weekly game time, and the highest score of IGDS9-SF, followed by adolescents with IGD but did not endorse negative consequences, adolescents without IGD but endorse negative consequences, and adolescents without IGD did not endorse negative consequences. Adolescents without IGD who endorse negative consequences reported the highest academic record, while adolescents with IGD who endorsed negative consequences reported the lowest academic record.

## Discussion

This study found that the 12-month prevalence of IGD was 4.6% among Chinese adolescents, and the prevalence of IGD among boys was significantly higher than that among girls. In addition, consistent with other studies, the students classified as having IGD by the DSM-5 criteria reported more gaming time and worse academic performance than those classified as not having IGD (5).

Agreement upon the criteria would allow the establishment of correct diagnoses to identify game players who need professional support and effective treatments (20). Our results demonstrated significant differences in the relationship between specific DSM-5 criteria and IGD diagnosis in the context of Chinese culture. The criterion “give up other activities” was the most relevant to the diagnosis of IGD based on the DSM-5, which is in accordance with Rehbein et al. (5). This criterion reflects increased priority given to gaming over other activities, implying behavioural salience, and is viewed as an essential feature of addictive behaviour (5, 21). In our study, if this criterion was endorsed, the probability of meeting the DSM-5 IGD criteria increased from 2 to 50%. “Negative consequences,” which reflects serious impairment of social function, was the next most relevant criterion to the IGD diagnosis (22–24). Among the adolescents who endorsed both “give up other activities” and “negative consequences,” 80% were classified as having IGD. “Continuation” also reflected the perceived negative consequences of gaming behaviour and was the third most relevant criterion to the IGD diagnosis (21). For those who additionally endorsed “continuation” to “give up other activities” and ‘negative consequences,” the probability of being classified as having IGD was increased to 93%. “Loss of control” is also viewed as an essential feature of addictive behaviour, and it is a necessary diagnostic criterion in the ICD-11 framework (10, 21). Although this criterion did not provide more information in the multivariate analysis, it had high predictive power for IGD independently.

Our results support the views of most clinical experts and researchers in this field. Most recently, a Delphi study conducted by Castro-Calvo et al. reported that the criteria of “give up other activities,” “continuation,” “loss of control” and “negative consequences” had the highest approval rate among international experts for their high diagnostic validity, while other criteria had the low approval rate (25). The present study provides evidence for inclusion of these criteria by the DSM framework in the future and supports the definition of gaming disorder in the ICD-11 framework.

The criteria of “tolerance” and “withdrawal” were of key importance for identifying IGD in the study of Rehbein et al. (5), however, these two criteria did not provide more information in the multivariate analysis in this study. This might be due to cultural differences. Although previous study has proven the adequate diagnostic accuracy of these two criteria (9), there are also doubts about whether these two criteria can distinguish between unpathological high involvement, such as a “gaming passion,” and problematic gaming (26).

The criteria of “preoccupation” and “escape” have high support rates, but the ability to predict IGD is weak, which is consistent with Rehbein's study (5). We also found that criterion “deception” showed a weak ability to predict IGD, which matches findings from studies in different cultural contexts (9, 27, 28). Thus, these three criteria may have limited distinguishing ability between gaming disorders and non-pathological game participation cross-culturally. Preoccupation with gaming behaviour may relate to high engagement (29, 30), escaping a negative mood by playing games may reflect a coping style for emotions (29, 31), and deceiving others may be influenced by others' attitudes towards the game, but this behaviour is not necessarily pathological (29). Future research can explore the impact of the removal of “escape” and “deception” on the validity of the DSM-5 framework. Given that salience is one of the core features of addictive behaviours (32), “preoccupation” might be worth keeping, but a more clearer definition might be needed to increase its validity.

Theoretically, all adolescents with IGD should report impaired social function (10, 28). However, 2% of IGD adolescents in our study did not report that gaming activity often leads to jeopardisation or loss of an important relationship, job or an educational or career opportunity. However, 3% of non-IGD adolescents reported that gaming activity often causes negative consequences. This may be due to the possibility of false positives or false negatives on the one hand; on the other hand, it may be due to the different insight of adolescents on the consequences of their gaming activity. These results suggest that the criteria for IGD should be defined by more specific intensity and frequency thresholds in future clinical practise and research work, such as for severe events, one event was sufficient; for moderate events, events needed to be repeated 3 times a week or more (28, 30).

There were some limitations of the present study. First, the IGD was assessed using self-reports and not clinical interviews by trained professionals. Second, the sample was restricted to 12- and 19-year-old Chinese students, and other groups may have different patterns of criteria endorsement.

## Conclusions

In conclusion, our study found that the estimated 12-month prevalence of IGD was 4.6%. Furthermore, symptoms related to “give up other activities,” “negative consequences,” and “continue despite problems,” are the most relevant to an IGD diagnosis in this sample.

## Data Availability Statement

The data that support the findings of this study are available from the corresponding author upon reasonable request and with completion of data user agreement.

## Ethics Statement

This study was carried out in accordance with the Declaration of Helsinki. Ethical approval was obtained from the Ethics Committee of Jiangxi Mental Hospital of Nanchang University (No. 20190113). All students and their parents were fully informed the purpose of the study. After obtaining informed consent from parents, an electronic informed consent form was then obtained from each student.

## Author Contributions

TL, YL, and SX: contributed in conceptualising and designing the study, analysis and interpretation of data, drafting and revising the article, and final approval of the version to be published. DW, JG, MH, and XC: contributed in collecting, analysis, interpretation of data, drafting the article, and final approval of the version to be published. YS and QS: contributed in revising the article. All authors contributed to the article and approved the submitted version.

## Funding

This study was supported by the Hundred Talents Program funding from Zhejiang University.

## Conflict of Interest

The authors declare that the research was conducted in the absence of any commercial or financial relationships that could be construed as a potential conflict of interest.

## Publisher's Note

All claims expressed in this article are solely those of the authors and do not necessarily represent those of their affiliated organizations, or those of the publisher, the editors and the reviewers. Any product that may be evaluated in this article, or claim that may be made by its manufacturer, is not guaranteed or endorsed by the publisher.
